# Ultra-Structural Alterations in *Botrytis cinerea*—The Causal Agent of Gray Mold—Treated with Salt Solutions

**DOI:** 10.3390/biom9100582

**Published:** 2019-10-08

**Authors:** Khamis Youssef, Sergio Ruffo Roberto, Admilton G. de Oliveira

**Affiliations:** 1 Agricultural Research Center, Plant Pathology Research Institute, 12619 Giza, Egypt; 2 Agricultural Research Center, Londrina State University, 86057-970 Londrina, PR, Brazil; sroberto@uel.br; 3 Biological Sciences Center, Londrina State University, 86057-970 Londrina, PR, Brazil; admilton@uel.br; 4 Laboratory of Electron Microscopy and Microanalysis, Londrina State University, 86057-970 Londrina, PR, Brazil

**Keywords:** *Botrytis cinerea*, salts, ATP, ROS, mitochondrial membrane potential

## Abstract

Potassium bicarbonate (PB), calcium chelate (CCh), and sodium silicate (SSi) have been extensively used as antifungal generally recognized as safe (GRAS) compounds against plant pathogenic fungi. In this research, in *in vitro* tests, the radial growth, conidial germination, and germ tube elongation of *Botrytis cinerea* was completely inhibited at 0.3% of PB, SSi, and CCh. In *in vivo* tests, application of PB, SSi, and CCh completely inhibited the occurrence of gray mold incidence of inoculated ‘Italia’ grape berries at concentrations of 1.0, 0.8, and 0.8%, respectively. In order to investigate the detailed mechanisms by which salts exhibited antifungal activity, we analyzed their influence on morphological changes by scanning electron microscopy (SEM) and transmission electron microscopy (TEM) and also on reactive species of oxygen (ROS), mitochondrial membrane potential (MMP), and adenosine triphosphate (ATP) content. Defects such as malformation and excessive septation were detected on salt-treated hyphae morphology observed by SEM. The internal structure of conidia treated or not with salt solutions was examined by TEM. In treated conidia, most of the conidia were affected and cellular vacuolization and cytoplasmic disorganization was observed. For ROS accumulation, a higher increase was observed in fluorescent conidia in presence of PB, SSi, and CCh by 75, 68, and 70% as compared to control, respectively. MMP was significantly decreased after salt application indicating a loss of mitochondria function. Also, luminescence showed that *B. cinerea*-conidia treated with salts contained less ATP than the untreated conidia. The results obtained herein are a step towards a comprehensive understanding of the mode of action by which salts act as antifungal agents against *B. cinerea*.

## 1. Introduction

Worldwide grape production estimated to be about 74,276.583 tonnes and harvested area nearly 6,931.353 ha with a yield 107160 hg ha^−1^ [1]. *Botrytis cinerea*, the causal agent of gray mold disease, is the second most significant phytopathogenic fungus around the world [2] and can infect several fresh fruit and vegetables worldwide, including table grapes, leading to severe economic losses [3,4,5]. Synthetic fungicides are regularly an important approach to control this disease. However, fungicide resistance has been detected in *B. cinerea* exposed to fungicides applied in grape vineyards [6]. In addition, market and regulatory pressure regarding residues and concerns of environmental and human health are increasing. In this context, new management techniques are needed to be adopted. Various studies have documented the strong antimicrobial activity of generally recognized as safe (GRAS) compounds including salts against postharvest diseases of several commodities counting table grapes [3,4,7,8,9]. Also, our previous results concluded that potassium bicarbonate (PB), calcium chelate (CCh), and sodium silicate (SSi) were the most effective salts against gray mold of ‘Italia’ and ‘Benitaka’ table grapes under artificial and natural field infections [3,4]. The benefits of salts, as alternative control means, are their incredible accessibility, simplicity of managing and use, and low cost when contrasted with different GRAS compounds [10].

From a quality point of view, preharvest treatment of PB and CCh was significantly effective in reducing mass loss and did not harm the film-forming properties of the ‘Italia’ berry skin, and it seems that they did not change cosmetic properties either [3]. In ‘Benitaka’ table grape, SSi did not influence the mass reduction, soluble solids content, and color index as compared to the water control [4]. Unfortunately, the information about the mechanism of action of those promising salts is poor. The mechanism of action still remains fundamentally unexplored. Understanding the mechanism of action of alternative control means such as salts may help to optimize their use against plant pathogens, particularly those attacking fruit and vegetables. It is outstanding that mitochondria are the fundamental source of cellular adenosine triphosphate (ATP) and play a focal role in an assortment of cell forms [11]. Also, mitochondria are the major endogenous source of reactive oxygen species (ROS), whose accumulation can prompt oxidation of macromolecules, resulting in mtDNA mutations, maturing, and cell apoptosis [12,13]. In any case, little data is available about whether PB, CCh, and SSi can cause mitochondrial damage of *Botrytis* conidia.

In the present research, effect of PB, CCh, and SSi on berry infection, pathogen radial growth, conidial germination, and germ tube elongation was explored. In addition, several approaches were carried out to investigate the mechanism of action of those salts including scanning electron microscopy (SEM), transmission electron microscopy (TEM), reactive species of oxygen (ROS) generation, conidium mitochondrial membrane potential (MMP), and adenosine triphosphate (ATP) content.

## 2. Materials and Methods

### 2.1. Chemicals, Fungal Isolate, and Plant Materials

Potassium bicarbonate (PB, KHCO_3_ 99.7–100.5%) was purchased from Synth, SP, Brazil; calcium chelate (CCh, C_10_H_12_N_2_O_8_CaNa_2_·2H_2_O 98%) was provided from Vetec Quimica Fina, Brazil and sodium silicate (SSi, Na_2_O 18% + SiO_2_ 63%) was supplied by Dinamica, SP, Brazil. *B. cinerea* isolate (BC-UEL-1) used in this study was provided from the fungal collection in Londrina State University, Brazil [3]. The used strain was isolated from infected ‘Italia’ table grapes showing typical gray mold symptoms. Healthy table grapes (*Vitis vinifera* L. cv. Italia) were harvested at full ripe from a commercial vineyard located at Paraná (South Brazil), pooled together, sterilized in sodium hypochlorite solution (1%) and were used for *in vivo* experiments.

### 2.2. Effect of Salts on Gray Mold Disease

The effect of PB, SSi, and CCh on controlling postharvest gray mold *in vivo* was evaluated as described in He et al. [5] with some optimization. In particular, ‘Italia’ grape berries were selected for uniformity, washed, sterilized, and air dried at room temperature before inoculation. Berries were wounded once with a sterile nail at the equator portion, inoculated into the wound site with 5 μL conidial suspension of *B. cinerea* at the concentration of 10^6^ conidia mL^−1^ on each wound, and left to dry for 2 h. Then 5 μL of each salt solution at different concentrations (0.2, 0.4, 0.6, 0.8, and 1.0%) was added to the same wound sites. Berries treated with water served as control. Treated berries were placed in plastic boxes (20 × 13 × 10 cm) and incubated at 23 ± 1 °C and high humidity (90–95%). Each treatment contained three replicates with 30 fruits each. The entire experiment was repeated twice. The incidence of decay (infected wounds, %) and disease severity (lesion diameter, mm) were recorded after 4 days of incubation.

### 2.3. Effect of Salts on B. cinerea Radial Growth

To examine the effect of PB, CCh, and SSi on the growth of *B. cinerea*, mycelia plugs (5 mm in Ø) from the growing edge of 1-week-old cultures were placed in the center of Petri dishes including potato dextrose agar (PDA) amended with tested salts at final concentrations 0.0, 0.1, 0.2, and 0.3%. Salt solutions were filtered through an EPS 0.45 µm filter (Millipore Express, Merck Millipore Ltd., Cork Ireland). Each salt/concentration includes five plates as replicates and the whole experiment was repeated twice. The inoculated plates were incubated at 23 ± 1 °C and radial fungal growth (mm) of *B. cinerea* was recorded at 24, 48, 72, and 96 h of incubation. The percentage of reduction in colony diameter (CD) was calculated as

CD (%) = (dc − dt) / dc × 100(1)

where: 

dc = average diameter of linear growth in control

dt = average diameter of linear growth in salt-treatment

### 2.4. Effect of Salts on *B. cinerea* Conidial Germination and Germ Tube Elongation

The effect of PB, CCh, and SSi solutions on *B. cinerea* on conidial germination (%) and germ tube elongation (µm) was determined according to the standard protocol. Conidial suspension at final concentration 10^6^ conidia mL^-1^ was mixed with tested salts at final concentrations 0.0, 0.1, 0.2, and 0.3% amended with potato dextrose broth (PDB) medium. The mixture was incubated at 23 ± 1 °C for 8 h. About 200 conidia were examined for germination rate and germ tube length (Olympus microscope CX41, Japan). Conidia were considered germinated if the germ tube was equal to or greater than the diameter of the conidium [8]. Each treatment includes five replicates and the whole experiment was repeated twice. The percentage of reduction of germinated conidia was calculated as

Reduction (%) of germinated conidia = (gc − gt) /gc × 100(2)

where: 

gc = average germinated conidia in control

gt = average germinated conidia in salt-treatment

Also, the percentage of reduction of germ tube length was calculated as

Reduction (%) of germ tube length = (gtc − gtt) / gtc × 100(3)

where: 

gtc = average germ tube length in control

gtt = average germ tube length in salts-treatment

### 2.5. Scanning Electron Microscopy (SEM)

Plugs of *B. cinerea* (6 mm in Ø) were cut from cultures grown for 96 h in PDA at 23 ± 1 °C treated or not with PB, CCh, and SSi at 0.2% and placed in vials containing 3% glutaraldehyde and 2% paraformaldehyde in 0.1 M sodium cacodylate buffer (pH 7.2) at 4 °C. Samples were kept in this solution for overnight for fixation and were then washed with 0.1 M sodium cacodylate buffer (pH 7.2) for 10 min three times. Consequently, the samples were dehydrated in an ethanol series (30, 50, 70, 90, and 100%) for 10 min three times. Samples were critical point dried with CO_2_ (BALTEC CPD 030 Critical Point Drier) created with gold (BALTEC SDC 050 Sputter Coater) and observed in a FEI Quanta 200 SEM operating at 25.0 KV [14].

### 2.6. Transmission Electron Microscopy (TEM)

Conidial suspension of *B. cinerea* (10^6^ conidia mL^−1^) was mixed with PDB amended with PB, CCh, and SSi at 0.2% was incubated at 23 ± 1 °C for 8 h. PDB mixed with conidium suspension was served as a control. Samples were centrifuged at 6,000 rpm for 10 min and pellet was fixed in 3% glutaraldehyde and 2% paraformaldehyde in 0.1 M sodium cacodylate buffer (pH 7.2) at 4 °C. Samples were washed with 0.1 M sodium cacodylate buffer (pH 7.2) for 15 min three times. Subsequently, the samples were post-fixed in 1% osmium tetroxide in 1% cacodylate buffer, embedded in Araldite resin (Electron microscopy Sciences, Hatfield, PA, USA). Stained 0.5 µm sections of TEM blocks were used to select appropriate sections. Ultrathin sections (60 nm) were obtained by using a ultramicrotome (Leica Ultracut UCT, Solms, Hesse, Germany), transferred to 200 mesh grids, stained with Reynolds’ lead citrate solution, then analyzed and photographed using a transmission electron microscope (JEOL, JEM-1400, Japan) at a current HT 80 KV according to [15].

### 2.7. Effect of Salt Solutions on Reactive Species of Oxygen (ROS) Generation

The probe 6-carboxy-2′,7′-dichlorodihydrofluoresce in diacetate, di(acetoxymethyl ester) (Molecular Probes, Inc. Eugene, OR, USA) was used to assess the intracellular ROS production levels in *B. cinerea* according to the manufacturer’s protocol (C2938). One mL of *B. cinerea* conidium solution (at a concentration of 10^6^ conidia mL^−1^) was treated with deionized water (dH_2_O), PB, SSi, and CCh at 0.2%, incubated for 8 h and centrifuged at 6,000 rpm for 10 min. Conidia were washed with 10 mM phosphate buffer saline (PBS) saline (pH 7.0) and re-suspended in the same buffer containing 10 µM acetoxymethyl ester dye (dissolved in dimethyl sulfoxide, DMSO). The suspension was incubated in the dark at 25 °C for 1 h. The loading buffer was removed and cells were returned to PBS buffer for 1 h until acetoxymethyl ester cleavage. Conidia were examined under fluorescent microscope (filter set 09 Zeiss: excitation: BP450-490 nm, emission: LP515 nm) microscope (Zeiss Axio Scope A1, Germany). Each treatment includes three replicates and the whole experiment was repeated twice. Five fields from each slide were randomly chosen; the numbers of conidia stained by acetoxymethyl ester dye in each treatment were calculated. Image capture was accomplished with an AxioCam MRc5 (Carl Zeiss) camera using Axio Vision Rel.4.7.2 software (Carl Zeiss Imaging Solutions GmbH, Germany) with a microscopic field area of 0.9 mm. The ROS production level was calculated as [16]

RPL = number of fluorescing conidia/number of conidia present in field image × 100.(4)

### 2.8. Effect of Salt Solutions on Conidium Mitochondrial Membrane Potential (MMP)

Loss of the membrane potential (Δψm) is a hallmark for cellular impairment and was measured with the mitochondrial membrane potential detection MitoProbe™ JC-1 Assay Kit [MitoProbe™ JC-1 Assay Kit (M34152), Molecular Probes, Inc.], which contained a cationic dye (5, 5′, 6, 6′-tetrachloro-1, 1′, 3, 3′-tetraethylbenzimidazolylcarbocyanine iodide) that fluoresced red in the mitochondria of healthy cells, and green in the cytoplasm of cells in which the mitochondria membrane collapsed. The ratio red/green fluorescence was considered a measure of mitochondria integrity: higher in healthy cells and lower in impaired cells. One mL of *B. cinerea* conidium solution (at a concentration of 10^6^ conidia mL^−1^) was treated with dH_2_O, PB, SSi, and CCh at 0.2%, incubated for 8 h and centrifuge at 6000 rpm for 10 min. *B. cinerea* conidia were re-suspended in 1 mL PBS buffer and then were incubated for 5 min at 37 °C. For the control tube, 1 µL of carbonyl cyanide 3-chlorophenylhydrazone (CCCP) was added. Then, 10 µL of 200 µM JC-1 was added and samples were incubated at 37 °C for 30 min. Conidia were washed once by adding 2 mL of PBS, centrifuged 5 min at 1,000 rpm, and the supernatant was removed. Cell pellet was gently re-suspended in 500 µL of PBS. Each treatment includes three replicates and the whole experiment was repeated twice. A fluorescent microscope (Zeiss Axio Scope A1, Germany) with red (546/12 nm excitation and 575–640-nm emission)/green (450-490-nm excitation and 515-nm emission) excitation filter was used to analyze the differential distribution of the red and green forms of the dye. The ratio red/green fluorescence was considered a measure of mitochondria integrity (mitochondrial membrane potential (MMP). J-aggregates were detected with the red channel and the monomeric form with the green channel. Images were captured as described above.

### 2.9. Effect of Salt Solutions on Adenosine Triphosphate (ATP) Content

*B. cinerea* pelleted conidia (10^8^) were treated with dH_2_O, PB, SSi, and CCh at 0.2%, incubated for 8 h, and centrifuged at 10,000 rpm for 15 min. ATP from treated conidia was extracted with 50 µL of 2.5% trichloroacetic acid (Synth, SP, Brazil) for 3 h at 4 °C. After centrifugation at 10,000 rpm for 15 min, 10 μL of supernatant was diluted with 115 μL of ATP-free H_2_O (deionized water) and 125 μL of ATP-free 40 mMTris-Acetate buffer (pH 8.0) as reported by [17]. For determination of ATP amounts in the extracts, a luciferin/luciferase kit was used (ATP Determination Kit (A22066)) according to the protocol of manufacturer (Molecular Probes, Inc. Eugene, OR, USA). Ten μL of diluted sample was mixed with 90 μL of standard reaction solution from the kit. A standard curve was generated for a series of ATP concentrations and the luminescence was measured as arbitrary units. The luminescence emission by the reaction was then determined with a multi-mode detection microplate reader (SynergyTM HT, BioTek Instruments, Inc. USA) at different incubation time (5, 15, 30, and 60 min). Each treatment includes three replicates and the whole experiment was repeated twice. The results were expressed as content of ATP (µmoles/10^8^ cell).

### 2.10. Statistical Analysis

Data were subjected to ANOVA (one-way analysis of variance) using Statistica 6.0 software. Mean values of treatments were compared by using Fisher’s protected least significant difference (LSD) test and judged *p* ≤ 0.05 level. When applicable, percentage data were arcsine-square root transformed in order to normalize variance.

## 3. Results and Discussion

### 3.1. Effect of Salts on Gray Mold Disease

**Figure 1:**
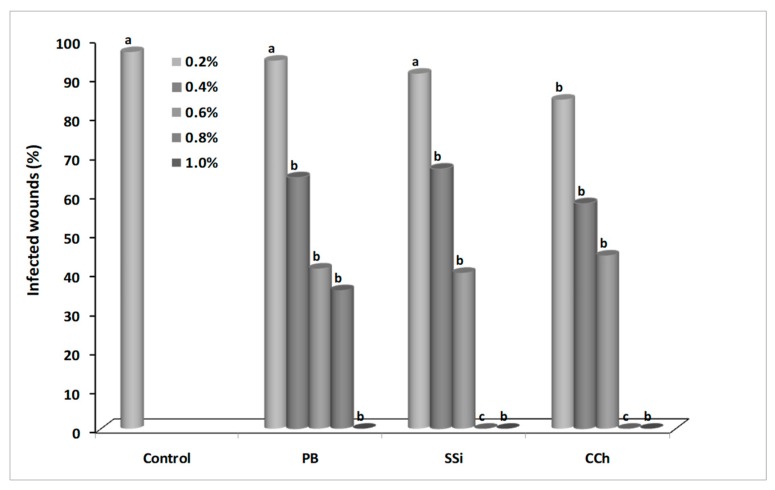
Decay incidence (% infected wounds) caused by *Botrytis cinerea* on ‘Italia’ table grapes untreated or treated with different concentration of potassium bicarbonate (PB), sodium silicate (SSi), and calcium chelate (CCh) after 4 days of incubation at 23 ± 1 °C. Values marked with the same letters are not statistically different by Fisher’s protected least significant difference test at *p* ≤ 0.05.

**Figure 2:**
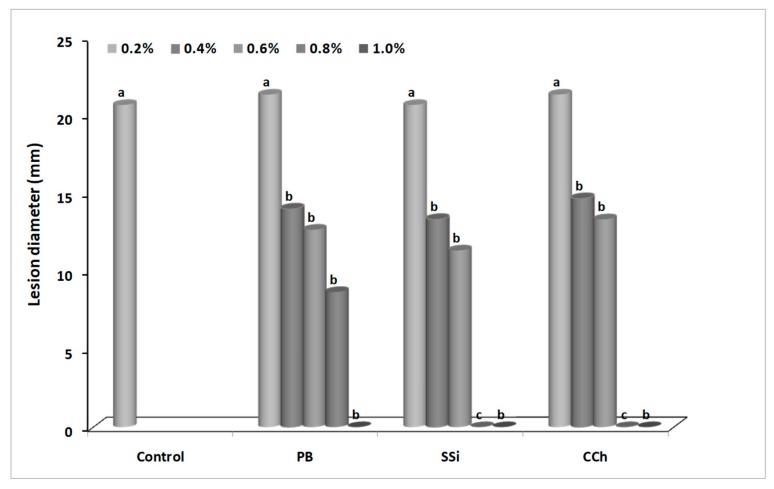
Decay severity (lesion diameter, mm) caused by *Botrytis cinerea* on ‘Italia’ table grapes untreated or treated with different concentration of potassium bicarbonate (PB), sodium silicate (SSi), and calcium chelate (CCh) after 4 days of incubation at 23 ± 1 °C. Values marked with the same letters are not statistically different by Fisher’s protected least significant difference test at *p* ≤ 0.05.

**Figure 3:**
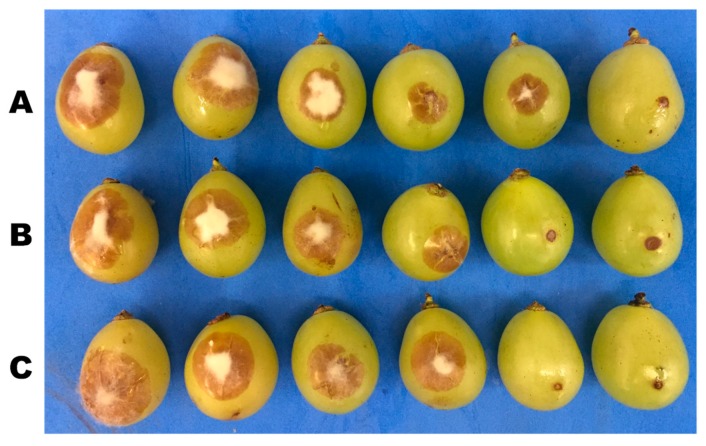
Effect of five concentrations of potassium bicarbonate (PB), sodium silicate (SSi), and calcium chelate (CCh) on gray mold disease of grapes. (**A**) Berries inoculated with *Botrytis cinerea* (10^6^ conidia mL^−1^) and treated with PB at 0.0, 0.2, 0.4, 0.6, 0.8, and 1.0% from left to right respectively. (**B**) Berries inoculated with *B. cinerea* conidial suspension and treated with SSi at 0.0, 0.2, 0.4, 0.6, 0.8, and 1.0% respectively. (**C**) Berries inoculated with *B. cinerea* conidium suspension and treated with CCh at 0.0, 0.2, 0.4, 0.6, 0.8, and 1.0% respectively.

Decay incidence and severity were recorded after 4 days of incubation. Generally, the efficacy of salts was positively correlated with the concentration. As concentration of salts increased, disease incidence decreased. For decay incidence and severity, application of 1.0% PB, 0.8% SSi, and 0.8% CCh completely inhibited gray mold incidence on grape berries (Figure 1 and Figure 2). For decay incidence, PB at 0.8% reduced the incidence of disease by 63% as compared to control. No significance differences were found between the three tested salts at 0.4, 0.6, and 1.0%. For decay severity, PB at 0.8% reduced the lesion diameter by 58% as compared to control. No significance difference was found between salts at 0.2% as compared to control (Figure 3). The results obtained herein confirm our previous ones on ‘Italia’ and ‘Benitaka’ table grapes [3,4]. Aqueous solutions (1.0%, w/v) of CCh, PB, and SSi reduced the occurrence of gray mold by 90, 80, and 89%, respectively as compared to the water control in immersion methods. In another study, a reduction of Botrytis incidence (23%) was obtained with sodium silicate as compared to control [7]. Qin et al. [8] concluded that decay incidence of gray mold was lower that 5% when 1% of boron was used. Recently, the effect of natamycin on inoculated grape berries was evaluated [5] and 50 mg L^−1^ reduced the lesion diameter of gray mold by 77% compared to control, while 100 mg L^−1^ completely inhibited the occurrence of mold.

### 3.2. Effect of Salts on *B. cinerea* Radial Growth

**Figure 4:**
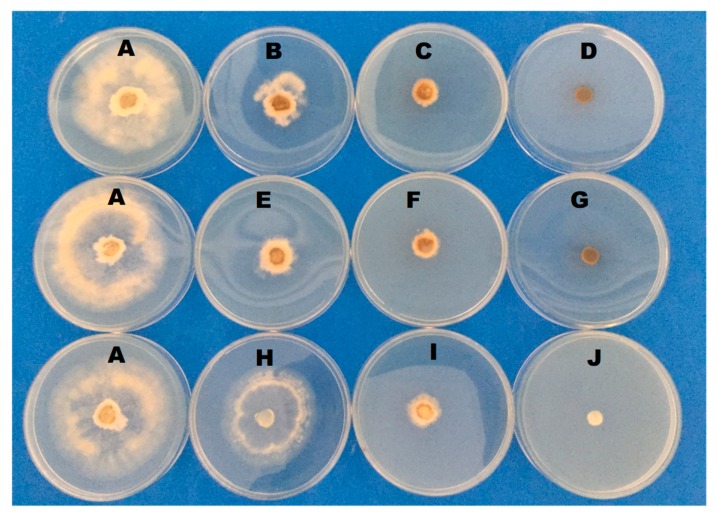
Effect of three concentrations of potassium bicarbonate (PB), sodium silicate (SSi), and calcium chelate (CCh) amended with PDA on the mycelial growth of *Botrytis cinerea* after 96 h of incubation at 23 ± 1 °C. (**A**) Control; (**B**–**D**) PB at 0.1, 0.2, 0.3% respectively; (**E**–**G**) SSi at 0.1, 0.2, 0.3% respectively; (**H**–**J**) CCh at 0.1, 0.2, 0.3% respectively.

The mycelial growth of *B. cinerea* was measured daily and the inhibition was positively correlated with the concentration of salts. The vegetative growth of *B. cinerea* was completely inhibited on the plate with 0.3% of PB, SSi, and CCh. After 96 h of incubation, the growth rate was decreased by 57 and 79% under the action of 0.1 and 0.2% PB; 65 and 80% of 0.1 and 0.2% SSi; 39 and 73% of 0.1 and 0.2% CCh, respectively (Table 1 and Figure 4). The results obtained herein confirmed other findings on antifungal activity salts against several plant pathogenic fungi. Youssef and Roberto [3,4] showed that PB, CCh, and SSi completely inhibited mycelial growth of *B. cinerea* at 0.25%. In the same way, sodium silicate at 100 mM completely suppressed the growth of *Trichothecium roseum in vitro* tests [18]. A significant reduction (up to 30%) of *Penicillium digitatum* growth was observed also in presence of sodium bicarbonate [16]. Also, Bi et al. [19] observed that the mycelial growth of *Alternaria alternata, Fusarium semitectum*, and *T. roseum* decreased significantly with increasing concentration of sodium silicate *in vitro*. Newly, the vegetative growth of *B. cinerea* was completely inhibited with 2 mg L^−1^ of natamycin on PDA plates [5].

### 3.3. Effect of Salts on *B. cinerea* Conidial Germination and Germ Tube Elongation

**Figure 5:**
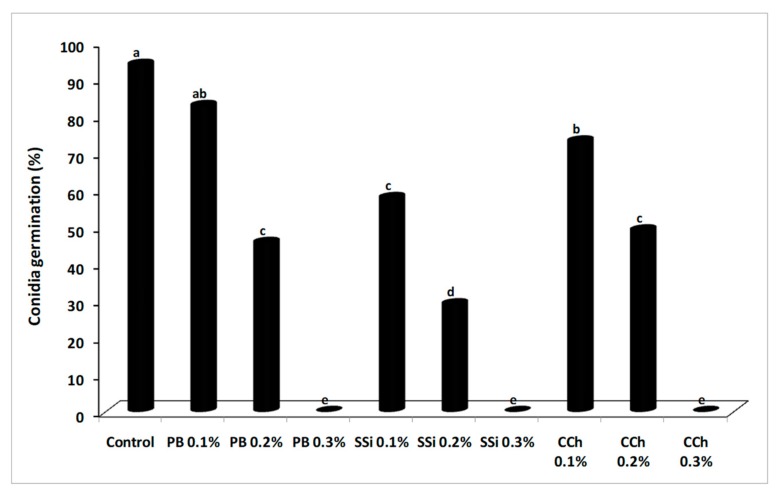
Effect of potassium bicarbonate (PB), sodium silicate (SSi), and calcium chelate (CCh) on conidial germination (%) of *Botrytis cinerea* in potato dextrose broth after 8 h of incubation at 23 ± 1 °C. Values marked with the same letters are not statistically different by Fisher’s protected least significant difference test at *p* ≤ 0.05.

**Figure 6:**
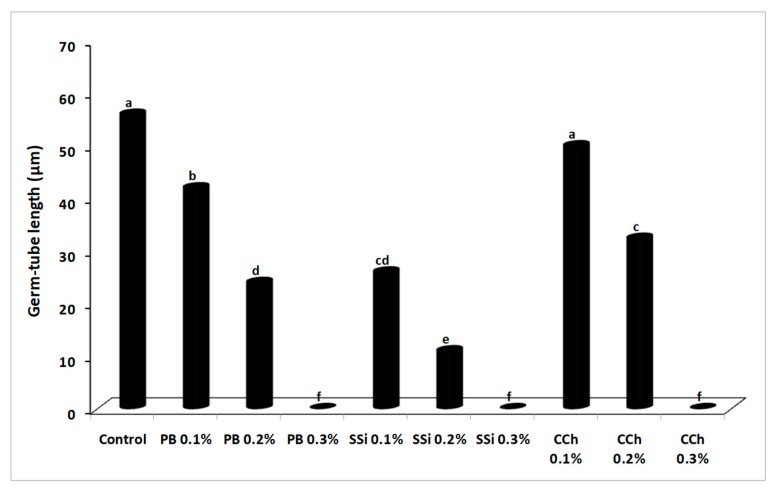
Effect of potassium bicarbonate (PB), sodium silicate (SSi), and calcium chelate (CCh) on germ tube elongation (µm) of *Botrytis cinerea* in potato dextrose broth after 8 h of incubation at 23 ± 1 °C. Values marked with the same letters are not statistically different by Fisher’s protected least significant difference test at *p* ≤ 0.05.

The inhibitory effect of salts on conidium germination and germ tube elongation of *B. cinerea* was shown in Figure 5 and Figure 6. Conidia germination and germ tube elongation were completely inhibited by PB, SSi, and CCh at 0.3%. After 8 h of incubation, the germination rate was decreased by 11 and 51% under the action of 0.1 and 0.2% PB; 38 and 69% of 0.1 and 0.2% SSi; 22 and 47% of 0.1 and 0.2% CCh, respectively (Figure 5). The direct activity of salts is well known [3,4,20,21,22,23,24,25,26,27,28,29,30] in inhibiting conidial germination, germ tube elongation, and production of pectinolytic enzymes in several pathogens. *B. cinerea* conidia immersed in solution of PB, CCh, and SSi at 0.25% completely inhibited conidia germination by 100% [3,4]. Several salts counting potassium tetraborate, calcium chloride, sodium carbonate, and sodium bicarbonate have been tested for their effectiveness to control gray mold, in small- and large-scale experiments [7,8,26]. Conidial germination and germ tube elongation were completely inhibited by boron at 0.5% and conidium germination was less sensitive to boron than germ tube elongation [8]. We noted that vegetative growth, conidial germination, and germ tube elongation of *B. cinerea* were more sensitive to SSi than PB or CCh at the same concentration. Our results were in agreement with Liu et al. [31] who demonstrated that conidial germination, germ tube elongation, and mycelial growth of *P. digitatum* were inhibited by sodium silicate at 0.1% (conidial germination) and 0.5% (mycelial growth). Also, sodium bicarbonate inhibited conidial germination of *P. digitatum* by 75% as compared to control and a similar effect was observed on germ tube elongation after 15 min of treatment [16]. In our study, a correlation was observed between results from artificial berry inoculations and *in vitro* tests, as salt treatments reduced disease incidence and severity, and inhibited pathogen growth and conidial germination. These results are in agreement with previous findings [3,4].

### 3.4. Scanning Electron Microscopy (SEM)

**Figure 7:**
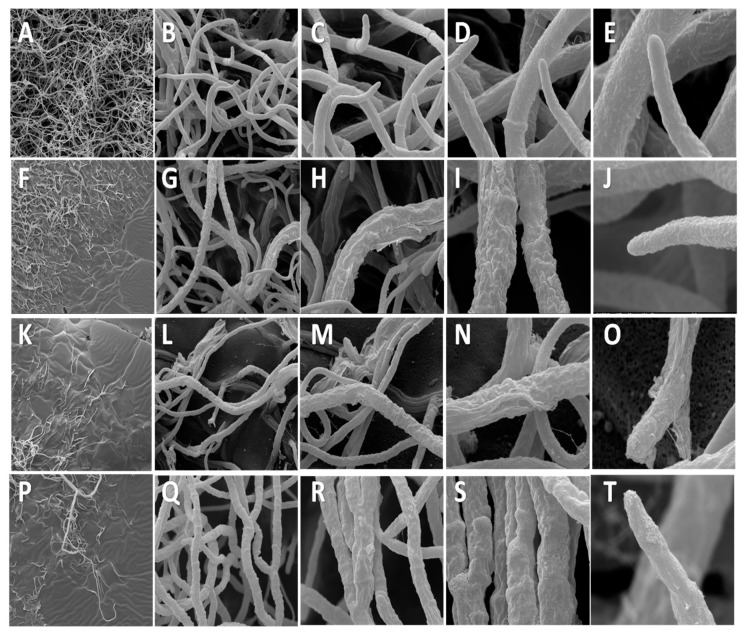
Scanning electron microscopy images of antifungal effect of salts against *Botrytis cinerea*. (**A**–**E**) Control after 96 h after incubation; (**F**–**J**) treatment with potassium bicarbonate (PB) at 0.2%; (**K**–**O**) treatment with sodium silicate (SSi) at 0.2%; (**P**–**T**) treatment with calcium chelate (CCh) at 0.2%. In the magnitude of 400× (A-P; bar 300µm), 3000× (B–Q; bar 20µm), 6000× (C-R; bar 20 µm), 12,000× (D-S; bar 10 µm) and 24,000× (E-T; bar 5 µm).

Morphological damage was detected in the hyphae treated with PB, SSi, and CCh compared to the hyphae in the control. Shriveled hyphae were commonly observed in salt-treated mycelia compared with the control-normal mycelia. Control demonstrating hyphae with typical ‘net’ structure and smooth surface while salt-treated hyphae lost their smoothness and formed unusual bulges on its surface. Defects such as malformation and excessive septation were detected on salt-treated hyphae morphology (Figure 7). SEM was previously used to demonstrate the morphology alternation of fungal hyphae, especially *B. cinerea* due to the treatment with various compounds. The direct examination by SEM indicated that the high concentration of nanoscale silicate (1000 mg L^−1^) could cause conidium and mycelia shrinkages of *B. cinerea* [32]. Also, silver nanoparticles caused Quercus wilting and damaged the mycelia of *Raffaelea* sp. [33] or *Colletotrichum acutatum* [34]. ZnO nanoparticles caused the defects for mycelia of *B. cinerea* and conidiophore of *Penicillium expansum* based on SEM inspection [35]. Similarly, SEM analysis showed that fludioxonil + metalaxyl-M caused inhibition of hyphal growth and defects on hyphae morphology of *Fusarium verticillioides* [36]. Recently, application of chitosan and silica nanoparticles caused asymmetrical branching of *B. cinerea* hyphae in the apical branch and the failure of linearity [37].

### 3.5. Transmission Electron Microscopy (TEM)

**Figure 8:**
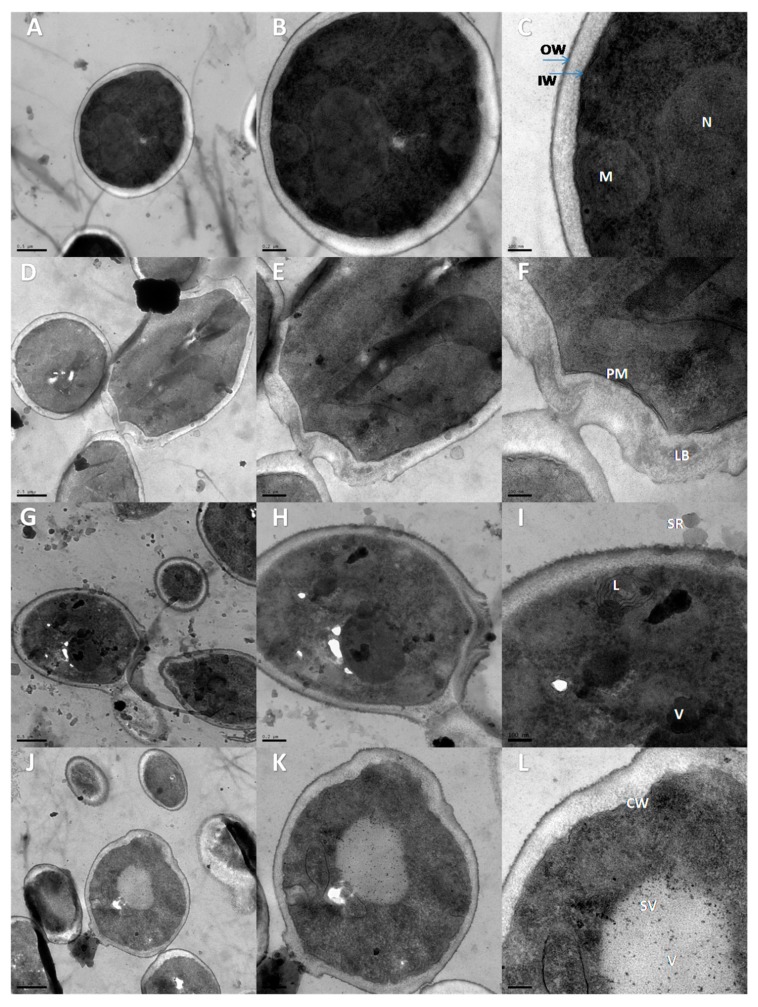
Transmission electron microscopy images show the internal structure of *Botrytis cinerea* conidia treated or not with salt solutions. (**A**–**C**) Control after 8 h after incubation; (**D**–**F**) treatment with potassium bicarbonate (PB) at 0.2%; (**G**–**I**) treatment with sodium silicate (SSi) at 0.2%; (**J**–**L**) treatment with calcium chelate (CCh) at 0.2%. In the magnitude of 20k (A, D, G, J; bar 0.5 µm), 40k (B, E, H, K; bar 0.2 µm), 80k (C, F, I, L; bar 100 nm). M: mitochondria, N: nucleus, LB: liposome-like bodies, L: lomasome, SV: spheric vesicles, IW: inner wall layer of conidium, OW: outer wall layer, V: vacuole, PM: plasma membranee, CW: cytoplasmic withdrawal.

The images produced by TEM showed the internal structure of *B. cinerea* conidia treated or not with salt solutions at 0.2% (*w*/*v*) (Figure 8). In untreated conidia (Figure 8A–C), conidia presented normal ultra-structures. In particular, normal cell, cytoplasm, and mitochondria were obvious and appeared normal in morphology. Also, the nuclei were visible with their well-defined envelopes. Conidium outer and inner cell wall showed no defects and was well-organized. In treated conidia, generally, most of the conidia were affected and cellular vacuolization and cytoplasmic disorganization was observed. When the conidia were treated with PB (Figure 8D–F), plasma membrane was fragmented and broken in many places and plasmolysis state showing the ruptured plasma membrane. Also, abnormal distribution and degradation of mitochondria in PB-treated conidia was noted. In SSi-treated conidia (Figure 8G–I), the cytoplasm was retracted and the cell wall was thinner than normal. Compared to untreated conidia, no recognized cellular structures were visible and cytoplasm was disorganized and withdrawn. In case of CCh-treated conidia, the dispersed cytoplasm was mixed with empty areas or vacuoles (Figure 8J–L). Also, the content of conidia appeared degraded and degradation of mitochondria was observed. In addition, severe void spaces were visible in the cell periphery and numerous vacuoles and spheric vesicles were observed. It was noted that the cell wall was opened and cytoplasm leakage was found out of the cell.

Based on the results obtained by TEM, it is believed that salts act directly on the conidia of *B. cinerea* leading to mitochondrial damage and reducing the infection rate of the fungus. It is well known that the mitochondrial respiratory chain is the major endogenous source of ROS, particularly when the mitochondria are damaged [38]. Correspondingly, when *B. cinerea* conidia treated with pterostilbene, disorganization, and coagulation of the cytoplasm and its withdrawal from the conidium wall, as observed by Pezet and Pont [39]. Our results are in agreement with those of Avis et al. [40] who detected abnormal mitochondria in *Fusarium sambucinum* and *Heterobasidion annosum* conidia treated with aluminum chloride and sodium metabisulfite, and included membrane retraction, undulation, and invagination. Therefore, it suggests that mitochondrial degradation of fungal conidia may be one of the important modes by which antifungal compounds inhibit fungal growth. Similarly, TEM was used to observe the plasma membrane disruption and cytoplasmic and mitochondrial disorganization of *Geotrichum citri-aurantii* and *Aspergillus niger* conidia treated with essential oils [41,42].

### 3.6. Effect of Salt Solutions on Reactive Species of Oxygen (ROS) Generation

**Figure 9:**
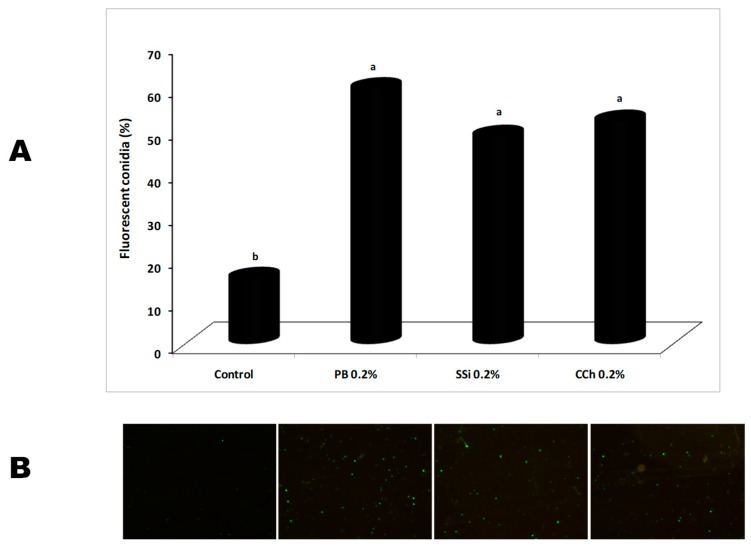
(**A**) Effect of potassium bicarbonate (PB), sodium silicate (SSi), and calcium chelate (CCh) at 0.2% on intracellular reactive species of oxygen (ROS) accumulation in *Botrytis cinerea*. ROS production level expressed as fluorescent conidia (%). Values marked with the same letters are not statistically different by Fisher’s protected least significant difference test at *p* ≤ 0.05. (**B**) Representative images of conidia from different treatments stained with acetoxymethyl ester probe under fluorescence microscopy were showed with a microscopic field area of 0.9 mm.

Reactive species of oxygen accumulation in *B. cinerea* conidia due to PB, SSi, and CCh was detected in terms of fluorescence emission, and the results showed that a higher number of fluorescent conidia was observed in presence of salt treatment as compared to the control (Figure 9). A higher increase in fluorescent conidia was observed in presence of PB, SSi, and CCh by 75, 68, and 70% as compared to control, respectively. Statistically, there was no significant different among the effect of salts itself. A considerable raise of ROS in treated *P. digitatum* conidia treated with electrolyzed sodium bicarbonate was recorded [16] which confirm our results. ROS could be in charge for pathogen inactivation and direct oxidation at the anode surface of organic compounds [43]. Also, under special stress conditions, high quantities of intracellular ROS are produced [44]. The production of ROS including superoxide anion and hydrogen peroxide can lead to death of the pathogen, by interfering with the pathogen life cycle, and causing numerous structural alterations. In presence of heat and H_2_O_2_, an increased production of ROS in *Monilia fructicola* and *Penicillium expansum* conidia, respectively was observed [45,46]. In addition, borate application stimulates ROS accumulation in *Colletotrichum gloeosporioides* conidia and resulting in mitochondrial harm [47]. Similarly, relative ROS levels were increased when *B. cinerea* conidia were treated with tea tree oil or methyl thujate [48,49].

### 3.7. Effect of Salt Solutions on Conidium Mitochondrial Membrane Potential (MMP)

**Figure 10:**
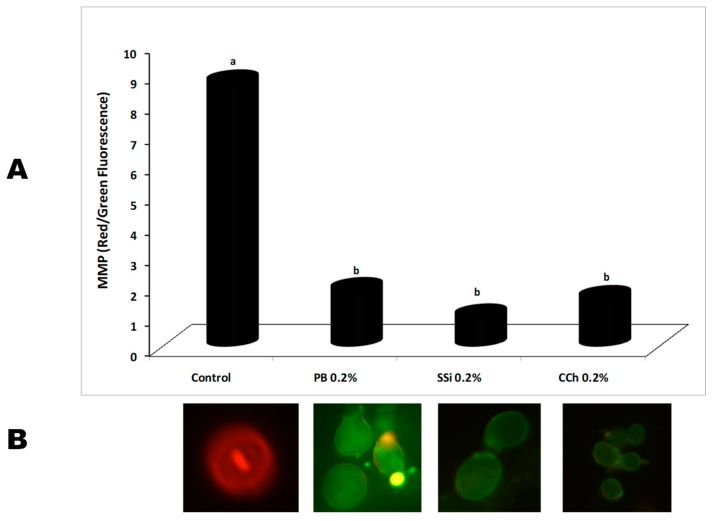
(**A**) Effect of potassium bicarbonate (PB), sodium silicate (SSi), and calcium chelate (CCh) at 0.2% on mitochondrial membrane potential (MMP) of *Botrytis cinerea* conidia represented as red/green florescence ratio and detected by MitoProbe™ JC-1 Assay Kit. Values marked with the same letters are not statistically different by Fisher’s protected least significant difference test at *p* ≤ 0.05. (**B**) For each treatment representative images of stained conidia under fluorescence microscopy were showed.

The ratio of red/green fluorescence is proportional to MMP, the higher the ratio, the more MMP in *B. cinerea* conidia. MMP was significantly decreased after salt application indicating a loss of mitochondria function. When *B. cinerea* conidia were treated with PB, SSi, and CCh the reduction of red/green fluorescence ratio reached 78, 88, and 81%, respectively. Also, no significant different among the salts was observed (Figure 10). It is well known that MMP is used as a marker of mitochondrial health, since this determine of ion transfer reveals metabolic action and integrity of the mitochondrial membrane. Our results obtained herein are in agreement with Fallanaj et al. [16] who demonstrated that the reduction of MMP was more than 61% on *P. digitatum* conidia exposed to electrolyzed sodium bicarbonate. The protection of MMP is linked with several mitochondrial functions especially ATP production [50]. Moreover, levels of mitochondrial dysfunction have been noted to closely follow intracellular ROS increase indicating some changes in mitochondria integrity and functionality [51]. Recently, He et al. [5] indicated that natamycin can damage the plasma membrane of *B. cinerea* and *P. expansum*, resulting the discharge of intracellular contents and eventual cell death.

### 3.8. Effect of Salt Solutions on ATP Content

**Figure 11:**
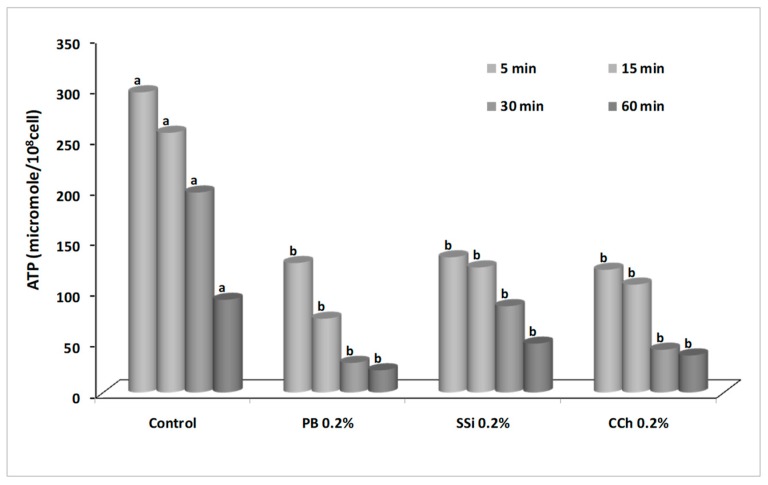
Effect of potassium bicarbonate (PB), sodium silicate (SSi), and calcium chelate (CCh) at 0.2% on ATP content of *Botrytis cinerea* conidia determined by luciferin/luciferase kit. The luminescence emission by the reaction was determined with a multi-mode detection microplate reader at different incubation time (5, 15, 30, and 60 min). Values marked with the same letters are not statistically different by Fisher’s protected least significant difference test at *p* ≤ 0.05.

The changes in ATP content were shown in Figure 11. Data showed that *B. cinerea* conidia treated with salts contained less ATP than un-treated control. Luminescence was measured after 5, 15, 30, and 60 min of incubation. After 15 min, ATP (µmoles/10^8^ conidia) was 265, 73, 123, and 106 for untreated conidia, PB, SSi, and CCh treated conidia, respectively. In particular, PB, SSi, and CCh reduced the ATP content by 72, 51, and 58%, respectively as compared to untreated conidia. Also, ATP content of conidia was decreased over incubation time. Obviously, mitochondria are the key basis of energy in eukaryotic cells, generating ATP throughout oxidative phosphorylation and the citric acid cycle [50]. Our results are in agreement with Fallanaj et al. [16] who confirmed that a decreased level of ATP in *P. digitatum* conidia treated by electrolyzed sodium bicarbonate was observed. Oxidative stress, associated with increased intracellular ROS, resulted in a mitochondrial membrane potential collapse, ATP decrease, and postponed growth [16]. Mitochondrial membrane permeability of *B. cinerea* cells was increased by tea tree oil application, as indicated by a decrease in intracellular ATP and an increase in extracellular ATP content [48]. Understanding the mechanism of action of salts will help to improve them to control postharvest decays and to disinfect fresh products which are derived by economic and consumer demand incentives. The results obtained on this study are mainly interesting for a successful marketable application of alternative control means such as salts to manage pre-postharvest gray mold of fruits and vegetables especially grapes.

## 4. Conclusions

In conclusion, our research showed that salts such as potassium bicarbonate, sodium silicate, and calcium chelate can alter the morphology and function of mitochondria in *B. cinerea*. Those salts caused accumulation of ROS, reduced ATP content, and decreased MMP resulting in the loss of mitochondria function. A correlation between results from artificial berry inoculations and *in vitro* tests was observed confirming parallels between *in vitro* and in planta conditions. The results obtained herein are a step towards a comprehensive understanding of the mode of action by which salts act as antifungal agents against *B. cinerea*. Host–pathogen transcriptome profiling could provide an additional useful tool to improve our knowledge about the direct and indirect effects of salt treatments on disease suppression.

## Figures and Tables

**Table 1:** Effect of three concentrations of potassium bicarbonate (PB), sodium silicate (SSi), and calcium chelate (CCh) amended with PDA on the mycelial growth of *Botrytis cinerea*.

Salts	Mycelial Growth (mm)			
	24 h	48 h	72 h	96 h
Control	19.2a	38.8a	60.8a	79.2a
PB 0.1%	13.2b	18.0c	29.2c	34.0c
PB 0.2%	10.8d	13.6e	14.8f	16.8f
PB 0.3%	0.0e	0.0f	0.0g	0.0g
SSi 0.1%	12.8bc	15.6d	23.2d	27.2d
SSi 0.2%	10.4d	12.8e	14.4f	15.6f
SSi 0.3%	0.0e	0.0f	0.0g	0.0g
CCh 0.1%	18a	32.8b	41.6b	48.0b
CCh 0.2%	11.6cd	18.8c	20.4e	21.2e
CCh 0.3%	0.0e	0.0f	0.0g	0.0g

Colony diameter was assessed daily at 23 ± 1 °C. Statistical analysis was performed within each column. Values marked with the same letters are not statistically different by Fisher’s protected least significant difference test at *p* ≤ 0.05.
